# Parents’ Awareness of Early Orthodontic Consultation: A Cross-Sectional Study

**DOI:** 10.3390/ijerph19031800

**Published:** 2022-02-05

**Authors:** Aljazi H. Aldweesh, Afnan A. Ben Gassem, Bashayer M. AlShehri, Adhwa A. AlTowaijri, Sahar F. Albarakati

**Affiliations:** 1 Division of Orthodontics, Department of Pediatric Dentistry and Orthodontics, College of Dentistry, King Saud University, Riyadh 11451, Saudi Arabia; salbarakati@ksu.edu.sa; 2 Department of Pediatric Dentistry and Orthodontics, College of Dentistry, Taibah University, AlMadinah AlMunawwarah 42353, Saudi Arabia; A_bengassem@hotmail.com; 3 College of Dentistry, King Saud University, Riyadh 11451, Saudi Arabia; Bashayer.J.94@gmail.com (B.M.A.); adwatwa9911@gmail.com (A.A.A.)

**Keywords:** parents’ awareness, orthodontic treatment, interceptive treatment, early diagnosis

## Abstract

Objective: The aim of this paper is to assess parents’ knowledge about early orthodontic consultation and treatment, and to determine the association of this knowledge with number of children, monthly income, children’s age and perceived dental problems. Methods: A questionnaire was distributed to 3000 school children aged 6–11 years. The children were asked to take the questionnaire to their parents and bring it back the next day. It consisted of 13 questions. Some of these questions were related to the gender of the child, number of children, and parents’ monthly income. The remaining questions assessed the parents’ awareness of their children’s need for orthodontic treatment. Results: In total, 2538 parents completed the questionnaire. Of these, 2014 (79%) of the parents thought that their children’s teeth would have a significant impact on their personality. Moreover, 1637 (64.5%) parents thought that their children had a problem with their teeth; 1080 (66%) of these parents consulted a dentist. Of these 1080 parents, 821 (76%) consulted an orthodontist, and of these 821 parents, 449 (55%) initiated the required orthodontic treatment. The number of children who visited an orthodontist was found to increase with an increase in age (*p* = 0.0057). Moreover, a perceived overjet was associated with a higher number of orthodontic consultations (*p* = 0.0326). Conclusion: Although parents’ awareness regarding their children’s orthodontic treatment is important, other factors, such as the age of the child, the severity of the malocclusion and the consulted dentist, play a role in initiating orthodontic treatment.

## 1. Introduction

Occlusal development begins in the sixth week of intrauterine life and continues until 24 years of age. The sequence of development proceeds in an orderly and timely manner, which is controlled by environmental and genetic factors [1]. An aesthetic occlusion is important for an individual’s self-esteem, attractiveness and acceptance among peers [2,3,4]. Children with malocclusion are reported to be teased, bullied and socially rejected, which may lead to psychological problems [2,3,4]. It is important to diagnose and manage the developing occlusion during primary, mixed and permanent dentition [1]. Early treatment (during the transitional period) can reduce the severity of the malocclusion and the complexity of the treatment [1]. Orthodontic treatment has different effects, including improved aesthetics, function and psychosocial wellbeing [5,6].

Although most patients referred to orthodontic professionals are children, the preadolescent stage is also important as dental development occurs during this period. Moreover, seeking proper management during this period is critical to achieve better dentofacial health and function. Parents play a vital role in their children’s orthodontic management [2,7]. They choose orthodontic treatment to improve their children’s oral health function and reduce social stigma [2,7]. Previous studies revealed that parents who have been former orthodontic patients or are willing to undergo orthodontic treatment are more approving of this treatment for their children [8,9,10]. Malocclusion is not considered a dental problem by most parents [11]. Many factors play a major role in determining parents’ perceptions and attitudes towards seeking orthodontic care for their children. These include the funding of orthodontic treatment, socioeconomic status, ethnic origin, availability of resources, literacy rate and knowledge on malocclusion [12]. Therefore, as a result of lack of knowledge and awareness, parents may not seek orthodontic treatment at the right time for their children. The aim of the present study is to evaluate parents’ knowledge about early orthodontic consultation and treatment and the association of this knowledge with their level of education, number of children, children’s age and perceived dental problems.

## 2. Materials and Methods

The present cross-sectional study protocol was approved by the Institutional Review Board (19/0067/IRB), King Saud University, College of Medicine, Riyadh, Saudi Arabia., before beginning the study. The questionnaire used in the present study was taken from a previous study conducted by Hassan et al. [13]. A pilot study on 30 parents was first carried out to assess its clarity. Results of the pilot study revealed that the questionnaire was easy to understand and parents did not face any difficulty filling it in.

The distribution of questionnaire was conducted using stratified random sampling, in which Riyadh was divided into 5 regions, middle, east, west, south and north, to avoid selection bias. The questionnaire was distributed randomly to 3000 children belonging to schools in these different regions in Riyadh, Saudi Arabia, between December 2019 and February 2020. The inclusion criteria for those included in the study were male and female students aged 6–11 years (according to schools’ records). The exclusion criteria were male and female students younger than 6 and older than 11 years of age, and students with craniofacial anomalies and syndromes.

The students were asked to take the questionnaire to their parents and bring it back the next day. It consisted of 13 questions. Some of these questions were related to the gender of the child “since females found to be more concerned about their appearance”, number of children “as it affects the parents’ knowledge and experience than parents with one child”, and parents’ monthly income (“orthodontic treatment is not free for every case, parents with higher monthly income will be able to pay for their children treatment”). The remaining questions assessed the parents’ awareness of their children’s oral health and need for orthodontic treatment. The outcomes were the parent’s knowledge of their children’s early orthodontic consultation, as well as their oral health. The exposures were monthly income, age, perceived malalignment and perceived overjet.

### 2.1. Sample Size

The sample size was calculated using the R statistical package, version 3.3.1 (20 December 2018, © 2018, R Foundation for Statistical Computing, Vienna, Austria) [14]. Cochran’s sample size formula for prevalence studies was used to determine the proper sample size [15]. The expected proportion of participants who consulted an orthodontist for their perceived dental problems was determined according to the study of Alnaafa et al. [16], assuming a proportion of 72.6%. A more conservative expected proportion of 30% was considered in the present study.

At a 95% confidence interval and 2% margin of error, the estimated sample size was 2017 participants. An additional 50% contingency for non-response increased the sample size to 3000 participants.

### 2.2. Statistical Analysis

Data regarding the responses to the survey questions are presented as frequencies and percentages. Pearson’s chi-squared test of independence was used to assess whether the responses to two questions were independent of each other. The result was verified at *p* ≤ 0.05. The data were considered to be statistically significant if the *p*-value was less than 0.05. The regression logistic model was applied using the stepwise method of variable selection to understand which demographic characteristics had an effect on consulting a dentist or an orthodontist and the treatment adherence. The concordance index, which is a standard measure of the predictive accuracy of a logistic regression model, was calculated for each model. The statistical package used in the present study was R statistical package, version 3.3.1 (20 December 2018, © 2018, R Foundation for Statistical Computing Vienna, Austria) [14].

## 3. Results

**Figure 1:**
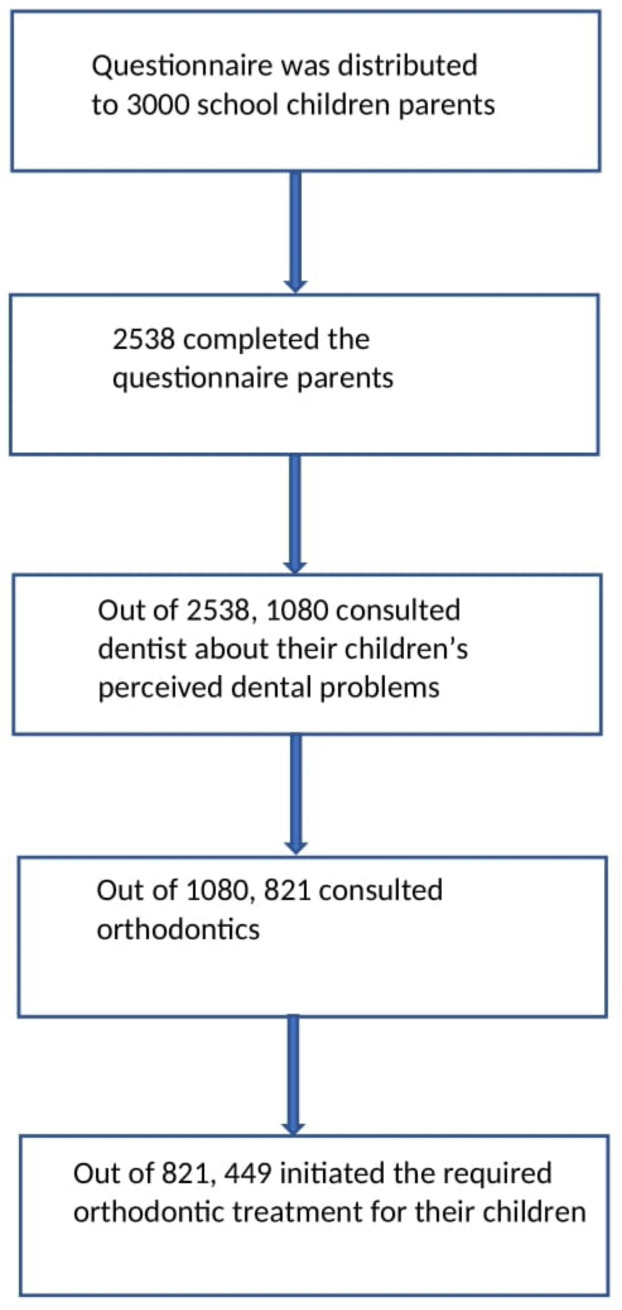
Flow diagram of the questionnaire distribution and respondent.

Out of the 3000 parents, 2538 completed the questionnaire (Figure 1).

Of these, 2414 (95%) were mothers and 149 (5%) were fathers. In total, 1374 (54%) of the children included in the present study were females and 1164 (46%) were males. Their mean age was 9.02 (±1.89) years (range: 6–11 years). Most children (860 (34%)) were aged 11 years. A total of 31% of the families had more than 4 children. Furthermore, 46% of the families had a monthly income between USD 2500–5000 (Table 1).

In total, 2014 (79%) of the parents thought that their children’s teeth would have a significant impact on their personality. While 1637 (64.5%) parents thought that their children had a problem with their teeth, 546 (33%) thought that their children’s teeth were not properly aligned; 1080 (66%) of these parents consulted a dentist regarding their children’s perceived dental problems. Of these 1080 parents, 821 (76%) consulted an orthodontist, and of these 821 parents, 449 (55%) initiated the required orthodontic treatment for their children (Table 2). No data was missing.

The association between the number of children per family and dental consultation was statistically insignificant (*p* = 0.6745), while the association between the monthly income of the family and dental consultation was statistically significant (*p* = 0.002) (Table 3).

The Pearson’s chi-squared test revealed that none of the demographic data had a significant association with orthodontic consultation (*p* > 0.05). Forty percent of the parents who initiated the required orthodontic treatment for their children after the orthodontic consultation had more than four children. There was a statistically significant association between the number of children per family and initiating the required orthodontic treatment (*p* = 0.0039). The assessment of the association of age and perceived dental problems with the orthodontic consultation revealed that the number of children who consulted an orthodontist increased with an increase in age (*p* = 0.0057); 391 (79.63%) of the children were aged 11 years when they consulted an orthodontist. In addition, a perceived overjet was associated with a higher number of orthodontic consultations (*p* = 0.0326). The remaining perceived dental problems were not associated with orthodontic consultations (Table 4).

Age and perceived dental problems were not associated with the initiation of orthodontic treatment (*p* < 0.05). Stepwise logistic regression analysis was performed to identify the predictors of dental consultation, orthodontic consultation and the initiation of the required orthodontic treatment. The results revealed that with each increase in the monthly income category of the family, the chance of consulting a dentist increased by 19%. With each increase in the age category, the chance of consulting an orthodontist increased by 11%. In addition, children with a perceived overjet were found to be 1.5 times more likely to consult an orthodontist than those without a perceived overjet. Children with perceived malalignment were found to be 1.25 times more likely to consult an orthodontist than those without perceived malalignment. The assessment of the predictors of initiating the required orthodontic treatment revealed that the chance of initiating the required orthodontic treatment increased by 16% with each increase in the age category. Moreover, the children with a perceived overjet were found to be 1.34 times more likely to initiate the required orthodontic treatment than those without a perceived overjet (Table 5).

## 4. Discussion

At present, preventive and interceptive treatment approaches play an important role in modern medicine. Interceptive orthodontics is defined as the phase of the science and art of orthodontics that is employed to recognize and eliminate potential irregularities and malpositions in the developing dentofacial complex [17]. Early orthodontic consultation is important for children. The American Association of Orthodontists states that the ideal time for a child to have his/her first orthodontic visit is at the age of 7 years [18]. Malocclusion can be caused by different factors, such as oral habits, dental anomalies and developmental position of the teeth. Periodontal problems, caries and temporomandibular joint problems can be caused by malocclusion [3]. Therefore, the awareness of malocclusion is extremely important. Most orthodontic patients are children and adolescents; therefore, their parents’ awareness of malocclusion is a very important factor influencing their motivation during orthodontic treatment [19].

In the present study, most parents believed that their children’s teeth would have a significant impact on their lives. This was in accordance with the findings of Hassan et al., [13] Alnaafa et al. [6] and Dann et al. [20], who reported that dentofacial appearance plays an important role in determining an individual’s attractiveness. In the present study, more than half of the parents 1637 (64.5%) reported that their children had a problem with their teeth; this was similar to the findings of previous studies [13,16,21]. Of these 1637 parents, 1080 (65.97%) consulted a dentist regarding the existing problems. Moreover, 821 (76.02%) of the 1080 parents consulted an orthodontist; this percentage was higher than that reported in previous studies [13,16]. This may be due to differences in the sample size.

The parents’ decision of initiating orthodontic treatment for their children is not merely their own and is affected by other factors, such as the dentist, speech therapist and other physicians [22]. Most (54.69%) parents who consulted an orthodontist reported that their children underwent orthodontic treatment. This was in accordance with the finding of Hassan et al.; in their study, 58.1% of the participants underwent orthodontic treatment [13].

In the present study, an association was noted between orthodontic consultation and the age of the child (*p* = 0.0057) and a perceived overjet (*p* = 0.0326); however, there was no effect of social class on orthodontic consultation. This was in accordance with the finding of a Finnish study, in which no association was noted between the orthodontic treatment and social class [23]. Meanwhile, King et al. reported that children with a high socio-economic status had a higher attendance for orthodontic consultations. However, their study had a low participation rate (143 (29%)) [24]. Kilpeläinen et al. reported that the parents of children with an overjet >7 mm were 5.5 times more likely to report that their children had been teased than those of children with a lesser overjet [25]. Dias and Gleiser found a relationship between crowding and increased overjet and orthodontic concern [26]. Similarly, we found that the parents of children with a perceived overjet were 1.5 times more likely to consult an orthodontist than those without a perceived overjet. The reason is that the increased overjet as well as crowding are at the anterior region, and this is usually associated with an unpleasing aesthetic [26].

In the present study, we found that the chance of consulting an orthodontist increased by 11% with each increase in the age category. Moreover, 79.63% of the children were aged 11 years when they consulted an orthodontist. The parents’ decision is important as the ability of a child to form his/her own opinion regarding orthodontic treatment is not developed until 10 to 11 years of age.

The present study has some limitations. The study included different parents belonging to different regions in Riyadh. In addition, the present study did not include questions assessing the parents’ knowledge about the right time to initiate orthodontic treatment. Future researchers should concentrate on evaluating parents’ knowledge about the right time to start orthodontic treatment, their knowledge about different types of orthodontic treatment options and awareness of the availability of different orthodontic appliances. The results of the current study cannot be generalized since a larger sample with parents from different regions and cities should be included.

## 5. Conclusions

In the present study, most parents believed that their children’s teeth would have a significant impact on their lives. The children’s age and a perceived overjet had an association with orthodontic consultation. Most (54.69%) parents who consulted an orthodontist reported that their children underwent orthodontic treatment. Children with a perceived overjet were found to be 1.5 times more likely to consult an orthodontist than those without a perceived overjet, while children with perceived malalignment were found to be 1.25 times more likely to consult an orthodontist than those without perceived malalignment. The orthodontic treatment predictors were age and a perceived overjet. With each increase in the age category, the chance of initiating the required orthodontic treatment increased by 16%. In addition, children with a perceived overjet were 1.34 times more likely to initiate the required orthodontic treatment than those without a perceived overjet. Although the parents’ awareness regarding their children’s orthodontic treatment is important, other factors, such as the age of the child, the severity of the malocclusion and the consulted dentist, play a role in initiating orthodontic treatment.

## Figures and Tables

**Table 1:** Demographic information and characteristics of participants in the study (*n* = 2538).

Question	Response	* n *	%
Q1: Relation to the child	Mother	2414	95.11
	Father	124	4.89
Q2: Gender of the child	Females	1374	54.14
	Males	1164	45.86
Q3: Age distribution of the children (years) Mean = 9.02 (SD = 1.89)	6	458	18.05
	7	206	8.12
	8	297	11.7
	9	303	11.94
	10	414	16.31
	11	860	33.88
Q4: Number of own children	1	216	8.51
	2	431	16.98
	3	528	20.8
	4	583	22.97
	>4	780	30.73
Q5: Household monthly income	Less than USD 2500	632	24.9
	USD 2500–5000	1165	45.9
	More than USD 5000	741	29.2

**Table 2:** Responses of the participants regarding the perceived dental and orthodontic needs and consultation.

Question	Response	*n*	%
Q6: Do you think that your child’s teeth would ever have a significant impact on his/her personality? (*n* = 2538)	Yes	2014	79.35
	No	524	20.65
Q7: Do you think your child has some problem with the positioning/alignment/symmetry of his/her teeth? (*n* = 2538)	Yes	1637	64.5
	No	901	35.5
Q 8: If YES, what problem is it? (*n* = 1637)	You think that your child’s teeth are coming forward	532	32.5
	There are spaces between teeth	487	29.75
	Your child’s teeth are crooked/not in a proper position	546	33.35
	You think that a tooth or teeth is/are missing	90	5.5
	You think that your child has extra teeth	52	3.18
	You are not sure about the problem of your child’s teeth, but you think that his/her smile is not pleasing	329	20.1
Q9: If yes, have you ever consulted a dentist about it? (Count = 1637)	Yes	1080	65.97
	No	557	34.03
Q10: If yes, have you ever consulted an orthodontist about it? (Count = 1080)	Yes	821	76.02
	No	259	23.98
Q13: If yes, is your child going through with the required treatment? (Count = 821)	Yes	449	54.69
	No	372	45.31

**Table 3:** Assessing the association between the demographic information and dentist consultation in children with perceived dental problems (*N* = 1637).

Question	Response	Dentist Consultation				Pearson’s Chi-Squared Test of Independence		
		Yes		No		χ^2^	*p*-Value	Interpretation
		*n*	%	*n*	%			
Q4: Number of own children	1	67	6.2	36	6.46	2.33	0.6745	Insignificant association
	2	148	13.7	86	15.44			
	3	219	20.28	123	22.08			
	4	284	26.3	135	24.24			
	>4	362	33.52	177	31.78			
Q5: Household monthly income	Less than USD 2500	249	23.06	163	29.26	12.47	0.002	Significant association
	USD 2500–5000	486	45	257	46.14			
	More than USD 5000	345	31.94	137	24.6			

**Table 4:** Assessing the association between age and perceived dental problems with orthodontist consultation in children (*N* = 1080).

Question	Response	Orthodontist Consultation				Pearson’s Chi-Squared Test of Independence	
		Yes		No		χ^2^	*p*-Value
		* n *	%	*n*	%		
Age	6	81	75	27	25	16.46	0.0057 *
	7	52	66.67	26	33.33		
	8	62	63.27	36	36.73		
	9	98	78.4	27	21.6		
	10	137	76.11	43	23.89		
	11	391	79.63	100	20.37		
You think that your child’s teeth are coming forward	Yes	296	80	74	20	4.57	0.0326 *
	No	525	73.94	185	26.06		
There are spaces between the teeth	Yes	206	71.78	81	28.22	3.55	0.0597
	No	615	77.55	178	22.44		
Your child’s teeth are crooked/not in a proper position	Yes	296	77.69	85	22.31	0.77	0.3814
	No	525	75.11	174	24.89		
You think that a tooth or teeth is/are missing	Yes	47	72.31	18	27.69	0.33	0.5667
	No	774	76.26	241	23.74		
You think that your child has extra teeth	Yes	27	71.05	11	28.95	0.29	0.5916
	No	784	76.2	248	23.8		
You are not sure about the problem of your child’s teeth but you think that his/her smile is not pleasing	Yes	157	73.71	56	26.29	0.63	0.4286
	No	664	76.59	203	23.41		

* Significance level at *p*-value ≤ 0.05.

**Table 5:** Stepwise logistic regression.

Results of the Stepwise Logistic Regression and Final Model for Dentist Consultation Predictors			
Parameter	Odds Ratio	95% CI	*p*-Value
Household monthly income	1.19	1.03, 1.39	0.0198 *
Concordance index for the model		57.13%	
**Results of the Stepwise Logistic Regression and Final Model for Orthodontist Consultation Predictors**			
**Parameter**	**Odds Ratio**	**95% CI**	***p*-Value**
Age	1.1	1.02, 1.2	0.0141 *
Perceived overjet	1.5	1.09, 2.08	0.0134 *
Perceived malalignment	1.25	0.92, 1.72	0.1568
Concordance index for the model		58.42%	
**Results of the Stepwise Logistic Regression and Final Model for Starting the Required Orthodontic Treatment Predictors**			
**Parameter**	**Odds Ratio**	**95% CI**	***p*** **-Value**
Age	1.16	1.04, 1.3	0.0089 *
Perceived overjet	1.34	1, 1.79	0.0481 *
Concordance index for the model		57.34%	

* Significance level at *p*-value ≤ 0.05.

## Data Availability

Data supporting reported results are available upon request (Table A2).

## Appendix A

**Table A1:** Consent Form.

INFORMED CONSENT FOR QUESTIONNAIRE-BASED STUDYForm # KSU-REC 006QS-E King Saud University, Riyadh, Kingdom of Saudi Arabia	
**Research Project Title:**	Parents’ awareness of interceptive orthodontic treatment: a questionnaire study
**Name of Principal Investigator:**	Dr. Aljazi Aldweesh
**Name and Address of Institution:**	King Saud University
**Contact No:**	
Dear Participants,	
I would like to take this opportunity to ask if you are willing to take part in this questionnaire-based survey. Please answer the questions to the best of your knowledge. All the requested information in this study questionnaire will be treated as confidential. If you are willing to voluntarily participate in this study, please tick the appropriate box below and sign this form and you will be given a copy for your own records.	
Signed by:	
Investigator’s Complete Name:	
Study Designation:	
Signature:	
Date (dd/mm/yyyy):	
[ ] I agree to participate in this study survey, and to utilize the information for scientific research purposes.	
Signed by:	
Participant’s Name:	
Signature:	
Date (dd/mm/yyyy):	

**Table A2:** STROBE Checklist.

**Title and abstract**	**Item No**	**Recommendation**	**Page No**
	1	(*a*) Indicate the study’s design with a commonly used term in the title or the abstract	1
		(*b*) Provide in the abstract an informative and balanced summary of what was conducted and what was found	1
**Introduction**			
Background/rationale	2	Explain the scientific background and rationale for the investigation being reported	1 and 2
Objectives	3	State the specific objectives, including any prespecified hypotheses	2
**Methods**			
Study design	4	Present the key elements of the study design early in the paper	2
Setting	5	Describe the setting, locations and relevant dates, including the periods of recruitment, exposure, follow-up and data collection	2
Participants	6	(*a*) Give the eligibility criteria, and the sources and methods of selection of participants	2
Variables	7	Clearly define all outcomes, exposures, predictors, potential confounders and effect modifiers. Give the diagnostic criteria, if applicable	2
Data sources/measurement	8 *	For each variable of interest, give sources of data and details of methods of assessment (measurement). Describe comparability of assessment methods if there is more than one group	NA
Bias	9	Describe any efforts to address the potential sources of bias	2
Study size	10	Explain how the study size was obtained	2
Quantitative variables	11	Explain how the quantitative variables were handled in the analyses. If applicable, describe which groupings were chosen and why	NA
Statistical methods	12	(*a*) Describe all the statistical methods, including those used to control for confounding	3
		(*b*) Describe any methods used to examine the subgroups and interactions	3
		(*c*) Explain how missing data were addressed	NA
		(*d*) If applicable, describe the analytical methods taking account of sampling strategy	NA
		(*e*) Describe any sensitivity analyses	NA
**Results**			
Participants	13 *	(*a*) Report the numbers of individuals at each stage of study, e.g., numbers potentially eligible, examined for eligibility, confirmed eligibility, included in the study, completing follow-up and analyzed	3 and 4
		(*b*) Give reasons for non-participation at each stage	NA
		(*c*) Consider the use of a flow diagram	3
Descriptive data	14 *	(*a*) Present the characteristics of study participants (e.g., demographic, clinical, social) and information on the exposures and potential confounders	4
		(*b*) Indicate the number of participants with missing data for each variable of interest	NA
Outcome data	15 *	Report the numbers of the outcome events or summary measures	4–7
Main results	16	(*a*) Present the unadjusted estimates and, if applicable, confounder-adjusted estimates and their precision (e.g., 95% confidence interval). Make clear which confounders were adjusted for and why they were included	NA
		(*b*) Report the category boundaries when continuous variables were categorized	4–7
		(*c*) If relevant, consider translating the estimates of relative risk into absolute risk for a meaningful time period	NA
Other analyses	17	Report other analyses conducted, e.g., analyses of subgroups and interactions, and sensitivity analyses	NA
**Discussion**			
Key results	18	Summarize the key results with reference to the study objectives	7 and 8
Limitations	19	Discuss the limitations of the study, taking into account the sources of potential bias or imprecision. Discuss both the direction and magnitude of any potential bias	8
Interpretation	20	Present a cautious overall interpretation of the results considering the objectives, limitations, multiplicity of analyses, results from similar studies and other relevant evidence	7 and 8
Generalizability	21	Discuss the generalizability (external validity) of the study results	8
Other information			
Funding	22	Present the source of funding and the role of the funders for the present study and, if applicable, for the original study on which the present article is based	8

* Give information separately for the exposed and unexposed groups. **Note:** “An Explanation and Elaboration” article discusses each checklist item and presents the methodological background and published examples of the transparent reporting. The STROBE checklist is best used in conjunction with this article (freely available on the Web sites of PLoS Medicine at www.plosmedicine.org Medicine at http://www.annals.org/, and Epidemiology at http://www.epidem.com/ accessed on 4 January 2022). Information on the STROBE Initiative is available at www.strobe-statement.org.
